# Web-Based Patient Education in Orthopedics: Systematic Review

**DOI:** 10.2196/jmir.9013

**Published:** 2018-04-23

**Authors:** Tessa Dekkers, Marijke Melles, Bob Sander Groeneveld, Huib de Ridder

**Affiliations:** ^1^ Faculty of Industrial Design Engineering Delft University of Technology Delft Netherlands

**Keywords:** patient education as topic, health education, orthopedics, internet, humans, preoperative care, patient satisfaction

## Abstract

**Background:**

Patients with orthopedic conditions frequently use the internet to find health information. Patient education that is distributed online may form an easily accessible, time- and cost-effective alternative to education delivered through traditional channels such as one-on-one consultations or booklets. However, no systematic evidence for the comparative effectiveness of Web-based educational interventions exists.

**Objective:**

The objective of this systematic review was to examine the effects of Web-based patient education interventions for adult orthopedic patients and to compare its effectiveness with generic health information websites and traditional forms of patient education.

**Methods:**

CINAHL, the Cochrane Library, EMBASE, MEDLINE, PsycINFO, PUBMED, ScienceDirect, Scopus, and Web of Science were searched covering the period from 1995 to 2016. Peer-reviewed English and Dutch studies were included if they delivered patient education via the internet to the adult orthopedic population and assessed its effects in a controlled or observational trial.

**Results:**

A total of 10 trials reported in 14 studies involving 4172 patients were identified. Nine trials provided evidence for increased patients’ knowledge after Web-based patient education. Seven trials reported increased satisfaction and good evaluations of Web-based patient education. No compelling evidence exists for an effect of Web-based patient education on anxiety, health attitudes and behavior, or clinical outcomes.

**Conclusions:**

Web-based patient education may be offered as a time- and cost-effective alternative to current educational interventions when the objective is to improve patients’ knowledge and satisfaction. However, these findings may not be representative for the whole orthopedic patient population as most trials included considerably younger, higher-educated, and internet-savvy participants only.

## Introduction

### Background

Patient education is a valuable part of care that enables patients to be informed, active participants in their own treatment [1-3]. Traditionally, it is provided through face-to-face teaching methods by health care professionals (HCPs) [3-5]. These methods are often supplemented with written booklets or pamphlets [4,6], or multimedia channels such as audiotapes, digital versatile disc, and video [7,8]. However, as both internet access and the availability of health information on public websites increases, it is now common for patients to also use the internet to learn about health and illness [9]. People with orthopedic conditions such as osteoarthritis, rheumatic arthrosis, or trauma form no exception to this trend. Internet use among this group increases rapidly: 79% of patients had internet access in 2012, and among them, 23% in 2010 to 65% in 2012 had used the internet to research their orthopedic condition or upcoming treatment [10,11].

Patients themselves are positive about using the internet to find health information. They perceive online health information to produce health benefits and social benefits (eg, improved self-care behavior and better social support) in a manner that is easily accessible, cost-effective, and time-effective [12]. Reactions of HCPs, however, have been mixed. It is recognized that health information that is distributed online can incorporate unique features such as tailored information, multimedia, and interactivity to keep patients engaged with the educational material [12,13]. For example, McKay and colleagues incorporated interactive elements in their internet-based diabetes self-management support intervention by allowing patients to live chat with each other and HCPs [14]. That such elements can ultimately enhance the education’s effectiveness is demonstrated, for example, in the fields of breast cancer and general surgery: Web-based patient education increases patients’ knowledge and satisfaction [15,16], improves the physician-patient relationship [17], and creates awareness about health issues in the general population [18]. Despite these initial successes, concerns with Web-based education have been voiced in orthopedic practice as well. Most of these stress the poor quality of online health information, which is deemed overly commercialized and poorly readable even when produced by qualified HCPs [19-21]. Furthermore, despite increasing internet access in the population as a whole, clinicians fear the generalizability of previous findings to elderly patients who may be inexperienced with internet usage [13,17,22]. To acknowledge these potential downsides while meeting patients’ demands for online patient education, it is important to systematically examine and evaluate the effects of Web-based educational interventions that are currently in place.

This review follows the definition of Roter and colleagues [23] in defining educational interventions as “pedagogic interventions, verbal or written, with a knowledge-based emphasis designed to convey information.” This distinguishes educational interventions from behavioral and affective interventions that focus on shaping behavioral patterns and appealing to feelings and emotions, respectively. The core aim of educational interventions is knowledge acquisition by patients [8,18,19,22]. With knowledge, the patient can participate in decision making and build skills for self-care [20]. In this way, increased knowledge can result in better clinical outcomes and ultimately improve the patient’ quality of life [24].

### Web-Based Patient Education in Comparison With Traditional Patient Education

When evaluating Web-based patient education, it is inevitable to compare its effectiveness with that of traditional patient education. Therefore, the first aim of this review was to compare the effectiveness of Web-based patient education with the more traditional methods for patient education, such as face-to-face teachings or the use of print materials. To make an accurate comparison between the two, we will provide a brief overview of the effectiveness of traditional patient education as identified in previous systematic reviews below.

In orthopedic practice, positive effects following traditional patient education include increased knowledge regarding surgical procedures and the informed consent process, improved self-management skills, and reduced length of stay [25-28]. Yet, educational interventions are no more effective than other interventions such as attention control or physiotherapy [29]. Furthermore, clinical outcomes such as pain and functioning do not improve following patient education [26,28,30], just as patient education also does not decrease anxiety in a clinically meaningful way [26,28]. Finally, there is insufficient evidence currently available to determine the effect of education on patients’ empowerment and self-efficacy [26], and no systematic reviews have examined the effect of patient education on patient satisfaction. From these findings, we hypothesize the following:

Hypothesis 1: Web-based patient education interventions have a positive effect on patients’ knowledge, but not on anxiety or clinical outcomes.

### Web-Based Patient Education in Comparison With Generic Health Information Websites

As outlined earlier in this introduction, educational interventions are no longer the sole source of knowledge for patients, as an abundance of health information is also freely available on the internet. When patients make use of generic health information while included in the experimental arm of a Web-based patient education intervention trial, online health information forms a potential strong co-intervention [13]. Thus, to accurately evaluate Web-based patient education, it is important to not only compare its effect with that of traditional interventions but also with that of public health information websites. Therefore, the second aim of this study was to compare Web-based patient education interventions with health information websites.

Health information websites are often broader in scope than educational interventions, as they typically target the general population as well as patients, whereas patient education targets patients or other members of the health care system only [31]. This means these websites are also unlikely to involve HCPs, or make use of clinical measurements or other information about patients that is derived from the health care system. Furthermore, health information websites are generally not theory-based. In contrast, patient education interventions are often developed and implemented using various theoretical frameworks [32]. Although we recognize that use of theory in intervention development is varied and may be absent from some patient education interventions as well [31-33], embedment of theory in general does set apart educational interventions from generic health information websites. Therefore, we expect and hypothesize the following:

Hypothesis 2: theory-based, or professionally facilitated, Web-based patient education interventions perform better than generic health information websites.

### Review Objective

Concluding, promising results of Web-based patient education interventions have been reported, but a systematic review of Web-based patient education specifically for orthopedic practice has not yet been carried out. The effects of Web-based patient education can be evaluated in itself but should also be compared with other interventions currently in place: (1) to traditional patient education interventions that are theory-based or professionally facilitated but are provided through different channels (such as verbally, written, or by using multimedia) and (2) to publicly accessible, generic health information websites that share the same channel of information provision (the internet) but are generally not theory-based or professionally facilitated. The overall aim of this systematic review was to tackle these comparisons by examining the effects of Web-based patient education interventions on patients with orthopedic conditions as reported in controlled and observational trials in comparison with traditional patient education and health information websites. The questions that guided us in examining the comparative effectiveness were as follows: (1) “what are the effects of Web-based patient education on adult patients with orthopedic conditions?” and (2) “what are the effects of Web-based patient education in comparison with the effects of traditional patient education and generic health information websites?”

## Methods

### Protocol and Registration

This systematic review has been written according to the requirements of the Preferred Reporting Items for Systematic Review and Meta-Analyses statement [34,35]. The review’s protocol has not been published.

### Eligibility Criteria

We included peer-reviewed, controlled, and observational trials reported in English or Dutch that self-defined as studying the effects of patient education interventions delivered via an online environment, including mobile devices, websites, and online systems, to adult people with any orthopedic illness or condition and currently receiving treatment for such conditions. Following our definition of educational interventions, we excluded behavioral or affective interventions. These may include educational components but differ from educational interventions as they specifically target behavioral patterns or appeal to feelings or social relationships to change patients’ outcomes [23]. As our focus lay with studying interventions, we did not include studies that only discussed generic, not theory-based, not professionally facilitated health information websites and did not compare their effectiveness with Web-based patient interventions. No mandatory principal outcomes were defined for studies to be eligible for inclusion in the review. No restrictions on publication date were imposed in the search for eligible studies. However, in the final selection of studies, we excluded studies that were published before 1995 to ensure the review represented current evidence.

### Information Sources

Studies were initially identified by searching the electronic databases CINAHL, Cochrane Central Register of Controlled Trials, EMBASE, MEDLINE, PsycINFO, PubMed, ScienceDirect, Scopus, and Web of Science from September 1, 2015 to November 30, 2015. As an example, the search strategy for the PubMed database can be found in Textbox 1. Search strategies for the other databases are available in Multimedia Appendix 1. The search was repeated in September 2017 to ensure the latest evidence was included. This search strategy was complemented by reviewing the bibliographies of included studies to identify additional studies of interest. We contacted one author for a full-text copy of an eligible study that was subsequently provided to the review team. For all other articles, full-text copies were available, and no further contact with the original authors was made.

### Study Selection

The first author assessed the identified studies for eligibility by title and abstract. The predefined selection criteria were applied to full-text reports of potentially eligible studies primarily by the first author in discussion with two review authors (MM and HdR) until consensus was reached. A third review author (BSG) was available for arbitration, but this was not required.

### Data Collection Process

A structured data extraction sheet was employed to extract data from included studies. The data extracted included (1) Study characteristics (ie, author, year of publication, design, population, and timing of outcome measures); (2) Intervention characteristics (ie, content and duration of intervention and control intervention, total sample size, and sample sizes in separate conditions); (3) Patient characteristics (ie, sociodemographic variables, health status, and experience with internet); and (4) outcomes (ie, type of outcome measure, instrument, and effect). For each study, the effect of the intervention was coded as (1) significant result (positive + or negative −), (2) nonsignificant result (=); or (3) not reported (×).

To provide a structured overview of the components in each intervention, we employed Barak and colleagues’ [36] framework for internet-supported interventions. This framework provides guiding definitions for four components that make up a Web-based education intervention, including (1) Program content (educational or behavior change content), (2) Multimedia use (type of media used to convey program content), (3) Interactive online activities (activities offered to increase patient interest, understanding, and engagement), and (4) Guidance and supportive feedback (if and how patients can obtain automated or human support and feedback).

**PubMed search strategy for the identification of studies assessing the effects of Web-based patient education interventions for the adult orthopedic population.**

(internet OR “world wide web” OR online OR web-based OR “computer assisted” OR e-health OR network OR “web services”) AND (“patient education” OR “patient education as topic” [MeSH Terms] OR “consumer health informati*” OR “medical education” OR “health education” OR “health knowledge, attitudes, practice”[MeSH Terms]) AND (orthopedic* OR orthopaedic* OR “joint replacement” or “arthroplasty” OR “hip” OR “knee”) AND (Adult OR Aged) AND (Effect OR efficacy OR performance OR result OR outcome)

### Risk of Bias in Individual Studies

To appraise the risk of bias in included studies, data regarding reporting, external validity, internal validity, and statistical power were extracted independently by two review authors (TD and BSG) using a modified version of Downs and Black tool for assessment of methodological quality [37]. This tool was selected for its high internal consistency and reliability and its applicability to both randomized and observational studies [37,38]. In line with previous studies, the ambiguous item regarding statistical power was modified to indicate the presence of a statistical power analysis or sample group calculation by allocating 1 (present) or 0 (absent) points [39-41]. The range of the modified tool is 0 to 28, with higher scores indicating higher methodological quality. Studies were not excluded on the basis of their methodological quality; however, findings from medium- and poor-quality studies were given less weight in the qualitative synthesis than studies of high methodological quality.

### Synthesis of Results

We examined the effectiveness of Web-based patient education interventions by describing and comparing the characteristics and results of the included studies, as summarized in the structured data extraction sheet (*see Data Collection Process*) through qualitative synthesis [42]. No meta-analysis was attempted because of the small number of included studies and considerable variability in the outcome measures employed.

## Results

### Study Selection

**Figure 1:**
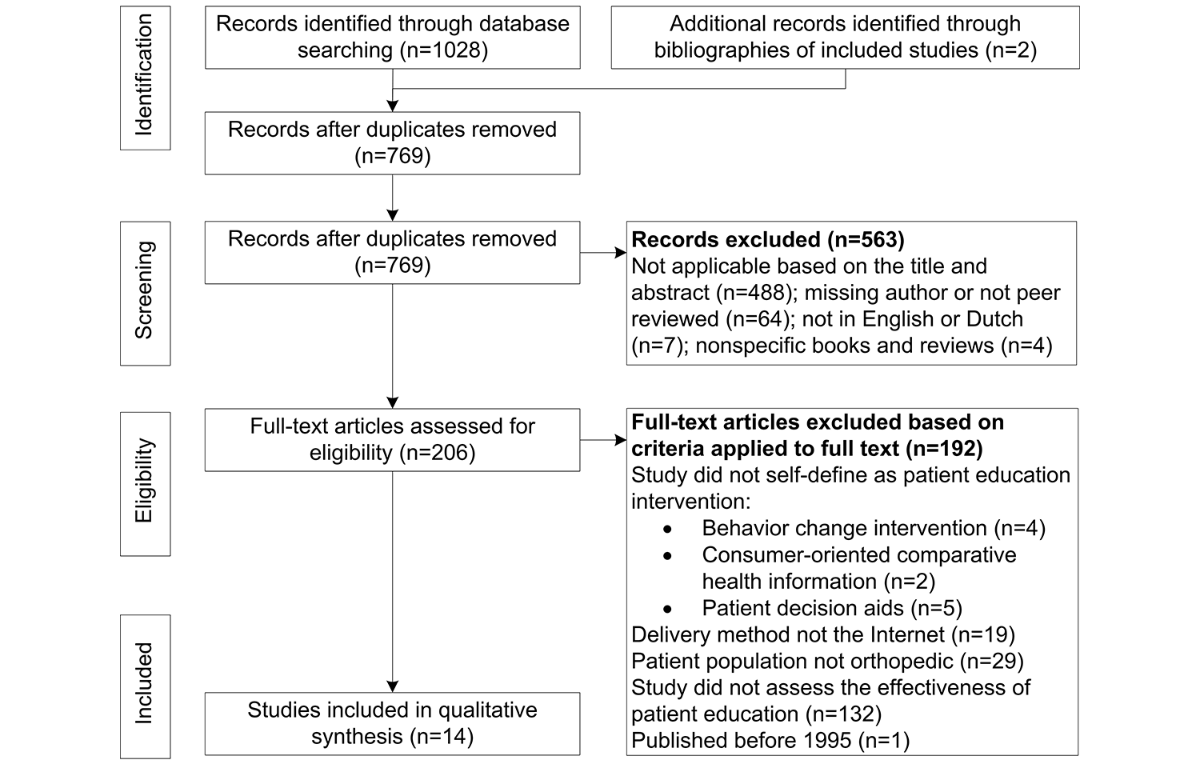
Preferred Reporting Items for Systematic Review and Meta-Analyses (PRISMA) flow diagram presenting identification and selection of articles for the systematic review of effectiveness of Web-based patient education in orthopedics.

The search identified 1032 eligible studies of which 10 trials, reported in 14 papers, met the inclusion criteria and were included in the review (Figure 1). Five of the included studies [43-47] concern separate reports of the same trial. To account for potential inconsistencies in reporting, all five reports of the trial were included in the review [35].

### Study Characteristics

Seven of the 10 trials employed a randomized controlled design, two an observational design, and one a quasi-experimental design. Four trials assessed the effect of Web-based patient education in comparison with traditional patient education channels, including face-to-face education with a nurse or physician [43-49] and patient information sheets [50]. Three trials compared Web-patient education with health information websites [51-53], and three assessed the interventions’ effects but did not compare these with either traditional patient education or health information websites [54-56].

### Patient Characteristics

Most of the studies provided Web-based patient education to patients undergoing surgical treatment, including total knee arthroplasty [48,50], total hip arthroplasty [48,50,55], knee arthroscopy [43-49], shoulder arthroscopy [43-48], anterior cruciate ligament reconstruction [48], and unspecified ambulatory orthopedic surgery [52]. 

Two studies provided Web-based patient education to patients with chronic conditions, including: rheumatoid arthritis [54] and osteoarthritis [56]. Two studies provided Web-based patient education to populations at risk for orthopedic conditions such as osteoporosis [51] and hip fracture [53]. The mean age of participants across studies was 56.3 years, and the sample was predominantly female (average 71.3% females in studies reporting gender). Most studies (70%) reported access to the internet as an explicit inclusion criterion, and some also required participants to also have an unspecified level of comfortableness [49] or skill [43-47,52,53] in using the internet.

### Intervention Characteristics

The intervention characteristics of all included studies are described in Multimedia Appendix 2. Most interventions consisted of a single website that was developed specifically for study purposes, whereas 1 study provided patient education by sharing multiple websites that are publically available [50]. We did not identify any studies that used mobile devices for patient education.

Program content was specific to each intervention. Most interventions offered practical information about the orthopedic condition or treatment, such as the procedures planned for the day of surgery or instructions for postoperative monitoring [43-50,52,55,56]. Others focused on providing information regarding behavioral determinants [51,52] and local health care services [54]. Only 2 studies explicitly reported using content that was not primarily educational: patient testimonials or narratives [53,56].

Half of the interventions conveyed content in a moderate to highly dynamic manner, meaning that they used three or more multimedia formats such as text, pictures, videos, animations, or audio [43-47,51,53,55,56]. The other interventions primarily used text and pictures to convey the content. We did not find consistent evidence for the obvious assumption dynamic multimedia use increases the intervention’s success. For example, both the static Orthoanswer website (primarily text) used by Fraval and colleagues [48] and the highly dynamic social cognitive theory website (text, graphics, audio, animation, and video) of Nahm and colleagues [56] increased patients’ knowledge. On the other hand, the similarly dynamic website of Drieling and colleagues [51] did not do so.

While half of the interventions could be considered dynamic in terms of multimedia use, only one provided highly dynamic activities (meaning, more than three interactive online activities were offered) [51]. Activities offered to the patient on the dynamic Bone Health Improvement Project website included problem-solving exercises, goal-setting exercises, and self-assessment. Among the more static websites, self-assessment was the most common interactive activity [53,56]. Due to the limited use of interactive online activities, we were not able to assess the influence interactivity might have on patient outcomes.

Most websites offered some human support or feedback as part of the intervention [43-47,51,53,54,56]. Examples of extensive support include a moderated message board [53] and highly tailored automated feedback [51,56]. Other interventions offered fairly limited support by only sharing contact details of a nurse or other health professional [43-47,54]. Again, there was no clear evidence that the level of support or feedback provided had an influence on the interventions’ success.

In terms of duration and frequency of website usage, we observed considerable variation. This ranged from single 20-min visits [49] to 18 repeated 60- to 90-min visits over the course of 6 months [51]. As duration and frequency were not consistently reported, we were not able to assess a dose-response relationship between usage of a Web-based intervention and outcomes.

### Methodological Quality of Included Studies

The methodological quality of the studies was moderate, based on a mean Downs and Black score of 17.67±5.42 out of 28 (Table 1) [37,38]. Most studies adequately reported intervention and sample characteristics, but the external validity was often problematic, as was the lack of power analyses.

### Outcome Measures of Included Studies

Most studies assessed knowledge acquisition [44,45,48-51,53-56] (90% of trials) and patient satisfaction, sometimes through qualitative feedback [46,48,49,51-53,55] (70% of trials). Other reoccurring outcome measures included anxiety [48-50], functional outcomes [45,52], and self-efficacy [46,51,53].

Many studies employed custom instruments that were designed by the researchers to assess the outcomes of their specific intervention. This resulted in a broad assortment of instruments that are difficult to interpret and compare (Table 2). To illustrate this diversity, consider instruments used to assess knowledge acquisition. Only one validated instrument (the Osteoporosis Health Belief Survey) was used in more than one study [51,53]. Four other studies also employed validated instruments, but not the same ones, as the topics of study (informed consent, anesthesia, and empowerment) differed considerably [44,48,50,56]. Four other studies employed instruments that had been developed specifically for each intervention, though the authors had pilot-tested or used these before [44,45,53,54]. Finally, 2 studies did not report anything regarding the validity or testing of their custom instruments [49,55].

### The Effects of Web-Based Patient Education Interventions in Orthopedics

A summary of the effects of Web-based patient education interventions is provided in Multimedia Appendix 3.

**Table 1:** Methodological quality of included studies (ordered by quality).

Study	Downs and Black [37] subscales^a^					
	Reporting	External validity	Bias	Confounding	Power	Overall study quality^b^
Heikkinen et al [44]	10	1	5	6	1	High
Fraval et al [48]	8	2	5	6	1	High
Drieling et al [51]	10	1	5	5	0	High
Nahm et al [53]	9	1	6	4	1	High
Heikkinen et al [43]	10	1	4	6	0	High
Heikkinen et al [47]	10	1	4	6	0	High
Heikkinen et al [45]	9	1	4	6	0	High
Umapathy et al [56]	10	1	5	3	1	High
Yin et al [49]	9	1	5	5	0	High
Groves et al [50]	6	1	6	5	1	High
Meesters et al [54]	9	1	5	1	0	Medium
Goldsmith and Safran [52]	7	0	5	2	0	Medium
Heikkinen et al [46]	5	1	3	3	0	Medium
Sobel and Popp [55]	3	0	1	0	0	Low
Median study quality	9/11	1/3	5/7	5/6	0/1	High

^a^Lowest to highest possible score for reporting (0-11), external validity (0-3), bias (0-7), confounding (0-6), power (0-1), and overall quality (0-28).

^b^Percentage scores were calculated by dividing the final score by the maximum score and multiplication by 100. The percentage scores were used for ordinal categorization of the studies as low quality (≤33%), medium quality (33.4%-66.7%), and high quality (≥66.8%) [38].

### Knowledge Acquisition

Web-based patient education significantly increased patients’ knowledge about orthopedic conditions and orthopedic treatment [44,45,48-51,53-55]. Web-based interventions were more effective than interventions provided through traditional channels [44,45,48-50], and these effects persisted over 2 weeks [45]. Increased knowledge levels also resulted in patients feeling more knowledgeable [44,45,49,54,55]. However, feelings of knowledgeability did not significantly increase more after Web-based education [44,45], except when provided in addition to face-to-face sessions [49].

Patients who received educational interventions did not acquire more knowledge than those who independently reviewed health information websites. One trial reported that a theory-based intervention produced higher knowledge levels regarding osteoporosis than a health information website in healthy older females [51], but another found no significant difference between both interventions in the same target group [53].

### Patient Satisfaction and Patient Feedback

Patient satisfaction was a main outcome in 2 studies [48,49]. Both found that Web-based patient education had a positive effect on patients’ satisfaction. Yin and colleagues report a persistent increase in satisfaction with information and teaching on the day of surgery (M_i_=8.7 vs M_c_=7.7, *P*=.03) and at the first postoperative visit (M_i_=9.2 vs M_c_=8.1, *P*=.01) after exposing knee arthroscopy patients to a custom online teaching module with explanations of anatomy, pathology, and perioperative instructions [49]. Fraval and colleagues report that satisfaction increased more in orthopedic outpatients who consulted both the online module and received verbal counseling with their surgeon compared with those who had only received the latter [48].

Seventy percent of trials investigated patient satisfaction or collected qualitative patient feedback but had not defined it as a principal outcome. Feedback on the online interventions was generally positive: patients described them as “very effective” [52], “easy to use” [43,50,55], and “worth the time” [49]. Compared with face-to-face education and health information websites, Web-based education was mostly evaluated better [48,53]. Only Heikkinen and colleagues report worse evaluations in terms of clarity of the content for the Web-based intervention (mean=79.75) compared with the face-to-face session with a nurse (mean=86.41), *P*=.001 [46]. However, both methods were considered clear enough to warrant further use.

### Anxiety

In the 3 studies that assessed patients’ anxiety following Web-based patient education, no significant effects on anxiety were found. Knee arthroscopy patients reported few distressing emotions in general, and anxiety was not influenced by Web-based patient education or verbal education [47]. After visiting a website providing an overview of the preoperative, intraoperative, and postoperative care processes, orthopedic outpatients were not less anxious about the planned surgery than patients who had discussed the same content with their surgeon [48]. For knee arthroscopy patients, using a Web-based educational tool did also not decrease anxiety about the surgery but did decrease anxiety about recovery [49].

**Table 2:** Patient outcomes and instruments used to assess the effect of Web-based patient education (alphabetical order).

Outcome measure	Instrument	Used in
Knowledge acquisition	Deaconess Informed Comprehension Test	[48]
	Hip Fractures Knowledge Test	[53]
	Knowledge Test	[44,45]
	Modified Standard Anaesthesia Learning Test	[50]
	Osteoporosis Health Belief Survey	[51,53]
	Orthopaedic Patients Knowledge Questionnaire	[44]
	Osteoarthritis Quality Indicator	[56]
	Sufficiency of Knowledge	[44,45]
	Custom instrument (no name provided)	[54,55,49]
Patient satisfaction and patient feedback^a^	Client Satisfaction Questionnaire	[48]
	Patients’ Evaluations of Education	[46]
	Perceived Health Website Usability Questionnaire	[53]
	Custom instrument (no name provided)	[49]
Anxiety	Emotions Questionnaire	[47]
	State-Trait Anxiety Index	[48]
	Patients’ Evaluations of Education	[46]
	Custom instrument (no name provided)	[49]
Empowerment, self-efficacy, and health attitudes	Calcium subscale of Osteoporosis Self-efficacy Scale	[53]
	Osteoporosis Health Belief Scale	[51,53]
	Outcome Expectations for Exercise Scale	[53]
	Patients’ Evaluations of Education	[46]
	Self-efficacy for Exercise	[53]
	Web-based Learning Self-efficacy Measure	[53]
Self-management and behavior change	Behavioral Risk Factor Surveillance System	[51]
	Block-National Cancer Institute Health Habits and History Questionnaire	[53]
	Brief Physical Activity Survey	[51]
	Health Education Impact Questionnaire	[56]
	Yale Physical Activity Survey	[53]
Clinical outcomes	The Symptoms	[43]
	Verbal Rating Scale of McGill Pain Questionnaire	[52]

^a^Qualitative feedback methods [51,52,55] are not included in the table.

### Empowerment, Self-Efficacy, and Health Attitudes

Two studies included self-efficacy as a primary outcome measure and reported contradicting evidence [51,53]. One study showed that both patients who used a structured social cognitive theory–based educational intervention and those who browsed health information websites had increased self-efficacy for calcium intake, the health behavior of interest [53]. In contrast, these effects were not replicated in a similar study that reported that self-efficacy was not influenced by patient education at all [51]. A lower quality report of the larger randomized controlled trial of Heikkinen and colleagues reported results that indicate that Web-based patient education may even adversely influence self-efficacy. When participants were asked how well they could act based on the knowledge received in the education, the intervention group perceived their abilities significantly lower (mean=82.77) than the control group (mean=88.86), *P*=.001 [46]. Thus, the extent to which Web-based educational interventions impact self-efficacy remains unclear.

### Self-Management and Health Behavior Change

Only one study assessed the effect of Web-based patient education on self-management [56]. In Umapathy and colleagues’ 2015 study, patients with self-assessed osteoarthritis used a tailored information tool to enhance self-management for 12 months. Users of the tool reported increased health-directed activity, engagement with life, self-monitoring, skill acquisition, and social integration but not significantly more so than nonusers. Users did acquire more knowledge about self-management and lifestyle as measured with the Osteoarthritis Quality Indicator and showed a significant reduction in weight (change score: −6.3%) compared with nonusers (change score: 2.5%), *P*= *.* 03. Although these results are promising, confounds in the study’s design contaminate its findings: participants in this study were not randomized to the conditions, and this opportunity for patients to self-select may have resulted in motivated users and demotivated nonusers.

### Clinical Outcomes

The evidence for an effect of Web-based patient education on clinical outcomes is limited and contradictory: although access to a pain management section of an ambulatory surgery website resulted in a significant decrease in “discomforting” pain scores after ambulatory surgery [52], Web-based tutorials about knee arthroscopy had no effect on pain after surgery [43]. In fact, the second study’s findings suggest that pain may be less effectively decreased after Web-based patient education in comparison with face-to-face education. Four weeks after the surgery, patients who had received Web-based education reported more pain in other areas (15.7% moderate-high pain) in comparison with the control group (7% moderate-high pain). However, three-way interactions between pain, group, and time failed to reach significance. The same study also reports that other postoperative symptoms (including tiredness, problems with digestion, and swelling of the operation area) decreased regardless of the patient education method used.

## Discussion

### Overall Findings

This review set out to examine the effects of Web-based patient education in the care for adult orthopedic patients. This is an important subject, as orthopedic patients are commonly using the internet to find health information [10,11] and perceive this to have an impact on both their health and social environment [12], although these effects have not yet been systematically examined. The comparative evaluation of Web-based educational interventions is especially relevant: to generic health information websites that potentially form a strong co-intervention [13] and to traditional patient education interventions that may be more effective but have higher costs [12].

This review identified 14 studies that reported the effects of ten different Web-based patient education interventions targeted toward the orthopedic patient population. Although the amount of studies is limited, the overall methodological quality of the included studies is high. Still, the different studies could not be compared on a meta-analytic level given the wide variety in scope, primary outcomes, and means of outcome assessment. Furthermore, the reported findings may be limited to patients who were already able to use the internet, as 70% of the studies included in this review established criteria that excluded inexperienced, less skilled patients with limited access to the internet to the trials. Hence, it is difficult to draw definitive conclusions about the effectiveness of Web-based patient education interventions.

While keeping these limitations in mind, the currently available evidence does suggest that patients who are offered Web-based patient education find the service both usable and satisfactory [43,48,49,51,52,53,55]. It increases their knowledge levels [44,45,48-50,53-55], which also results in patients who feel knowledgeable [44,45,49,54,55] and are able to participate in the informed consent process [48,49,55]. Web-based education appeared to be more effective in these aspects than traditional education methods [44,45,48-50]. Despite their knowledge gain, the provision of online information to patients does not subsequently reduce patients’ anxiety [47-49]. These findings support our first hypothesis that Web-based patient education interventions would have a positive effect on patients’ knowledge but not on anxiety. Contrary to second hypothesis, however, Web-based education was not found more effective than generic health information websites [49,51]. A possible explanation for this finding is that both Web-based patient education materials and generic health information websites suffer from issues such as poor readability [21,57-59].

There is still insufficient evidence to determine the effect of Web-based patient education on self-efficacy, self-management, or clinical outcomes. Only 2 studies investigated self-efficacy [51,53], 1 observational study investigated self-management [56], 2 studies investigated pain [43,52], and no studies have assessed patients’ functioning using standardized patient-reported outcome measures for orthopedic practice, such as the Western Ontario and McMasters Universities Osteoarthritis Index or Hip Disability and Osteoarthritis Outcome Score. Therefore, we were unable to test our hypothesis that Web-based patient education would not have an effect on clinical outcomes.

This review illustrates the typical Web-based patient educational intervention that is currently offered to people with orthopedic conditions. These are mostly websites focused on practical, informational content that is presented using multiple media formats including text, pictures, and video. Most offer some form of (human) support to patients using the programs but are still static in terms of interactivity. Still, it seems that online self-assessment is being recognized as an appropriate strategy to make educational content more engaging. At this point, there was not enough evidence to conclude that either of these intervention characteristics—content, media use, support, interactivity, or duration—has a consistent effect on the interventions’ success. However, regarding support provision, it should be noted that almost all studies that did not specify the level of support offered on the website did include some form of provider contact as part of the usual care given to both the experimental and control groups [48-50,52]. Patients may have received feedback and support during these meetings, which makes it difficult to estimate the effects that added online support or feedback may have. Therefore, future work should report whether (information and communication technology) support or feedback was provided as part of usual care.

Most of our findings are in line with previous reviews of Web-based patient education. We found further support for the idea that changing the channel of communication in patient education can increase patient satisfaction, as was tentatively hypothesized in Nguyen and colleagues’ 2004 review [13]. Web-based patient education is also equally effective in orthopedics as in oncology practice [15]. Similarly to orthopedic patients, breast cancer patients’ knowledge and satisfaction increased following Web-based education, whereas their anxiety was not affected. Furthermore, in both fields, a wide variety of study outcomes and corresponding instruments was identified. Thus, this review can only further endorse the need for standardized instruments in the evaluation of Web-based interventions as previously addressed by Ryhänen and colleagues in 2010 [15].

Despite the aforementioned replications, we could not determine whether self-care behavior of orthopedic patients increased because of Web-based patient education, an effect that has been identified in cardiovascular patients who were offered online educational interventions [60]. Because the internet can be used without constant professional supervision, online interventions may play a continuous role in the education and support of chronically ill orthopedic patients [61-63]. Despite this potential, we found only 1 study that specifically evaluated education within the context of an online self-management intervention [56]. This may have been because we have excluded behavioral or affective interventions from review. This narrow scope allowed us to precisely examine the effectiveness of education alone, but a next step for Web-based interventions would be report separately on educational, behavioral, and affective content. This will allow those who are tasked with developing interventions to study the interplay between these components to determine the “ideal” dose for a specific population or condition. Taxonomies to facilitate such in-depth examination of intervention components have already been developed for behavior change techniques [64] and computer tailoring [65]. Slowly, similar efforts are done for Web-based interventions as well, such as Barak and colleagues’ internet-supported interventions model [36] used in this review to describe intervention components and Win and colleagues’ online patient education features model [12]. Still, a consensus on an appropriate taxonomy has not yet been reached, and until this is in place, it will be difficult to estimate the specific role education can play in enhancing complex outcomes such as self-management capabilities.

### Limitations

This review has several limitations that relate to the representativeness of the samples included in the studies, the limited number of included studies, and the lack of a meta-analysis.

First, the quality of the reported studies was higher than what previous reviews of Web-based interventions have documented [13,66]. Most studies provided an elaborate description of the control groups and interventions, including the specific interactive elements designed into the programs. Still, the external validity of the included studies is low; no studies provided evidence that the included sample was representative of the entire population. This is concerning considering that most studies had criteria in place that excluded participants with less internet use and experience. Compared with these selected samples, the entire population was likely older [10,67-69], lower educated [10,68,69], and more likely to receive public care [10,70]. On the other hand, younger patients are also the ones who expect more information [71,72], value online services [73,74], and are most likely to benefit from educational interventions [75]. Thus, although we cannot conclude that it serves the whole orthopedic population, Web-based patient education may be an excellent way to cater to this younger patients’ specific needs.

Second, we were able to evaluate only a limited number of studies. Although the initial search identified over a thousand potential studies, only ten trials specifically evaluated Web-based patient education interventions in a sufficiently controlled setting. As a result, we were not able to draw any reliable conclusions about the effect of Web-based patient education on patient reported outcomes, including postoperative pain and functioning, whereas reviews of traditional patient education show that these outcomes may be affected [28-30].

Third, the studies employed a wide variety of outcome measures that did not allow for a meta-analysis of the findings. Though the qualitative synthesis does indicate that Web-based patient education increases patients’ knowledge levels and satisfaction, we were not able to determine the extent of these effects. Therefore, their clinical relevance has yet to be determined.

### Conclusions

In summary, offering patient education interventions via the internet to adult people with orthopedic conditions increases their knowledge about their condition and its treatment [44,45,47-51,53-55]. Online educational interventions are typically instructional websites that make use of multimedia but offer limited interactivity. They are considered usable and can increase patient satisfaction [43,48,49,50,52,53,55]. However, the provision of online information to patients does not subsequently reduce patients’ anxiety [47-49].

Given these findings, we tentatively conclude that Web-based patient education may be offered as a time- and cost-effective alternative to current educational interventions when the primary aim of the intervention is to increase patients’ knowledge and satisfaction. However, there is too little evidence to advocate for Web-based patient education to replace existing interventions that aim to improve other outcomes, including self-management skills, pain, and function. Furthermore, it should be kept in mind that Web-based interventions currently cater to younger patients who may not be comparable to the general patient population. A solution for hospital administrators or health care policy makers currently planning an educational intervention for orthopedics patients is to provide Web-based education in addition to verbal or written components, which allows patients to select the platform they are most comfortable with while ensuring satisfactory results.

## Appendix

Multimedia Appendix 1 Search strategies for the identification of studies assessing the effects of Web-based patient education interventions for the adult orthopedic population.

Multimedia Appendix 2 Intervention characteristics of studies evaluating the effects of Web-based patient education in orthopedics (alphabetical order).

Multimedia Appendix 3 Summary of the effects of Web-based patient education (ordered by comparison then by quality).

## Abbreviations

HCP: health care professional

## References

[1] Gruman J, Rovner MH, French ME, Jeffress D, Sofaer S, Shaller D, Prager DJ (2010). From patient education to patient engagement: implications for the field of patient education. Patient Educ Couns.

[2] Fernsler JI, Cannon CA (1991). The whys of patient education. Semin Oncol Nurs.

[3] Hoving C, Visser A, Mullen PD, van den Borne B (2010). A history of patient education by health professionals in Europe and North America: from authority to shared decision making education. Patient Educ Couns.

[4] Pellino T, Tluczek A, Collins M, Trimborn S, Norwick H, Engelke ZK, Broad J (1998). Increasing self-efficacy through empowerment: preoperative education for orthopaedic patients. Orthop Nurs.

[5] Lübbeke A, Suvà D, Perneger T, Hoffmeyer P (2009). Influence of preoperative patient education on the risk of dislocation after primary total hip arthroplasty. Arthritis Rheum.

[6] Cheung A, Finegan BA, Torok-Both C, Donnelly-Warner N, Lujic J (2007). A patient information booklet about anesthesiology improves preoperative patient education. Can J Anesth.

[7] Lin PC, Lin LC, Lin JJ (1997). Comparing the effectiveness of different educational programs for patients with total knee arthroplasty. Orthop Nurs.

[8] Daltroy LH, Morlino CI, Eaton HM, Poss R, Liang MH (1998). Preoperative education for total hip and knee replacement patients. Arthritis Care Res.

[9] Colledge A, Car J, Donnelly A, Majeed A (2008). Health information for patients: time to look beyond patient information leaflets. J R Soc Med.

[10] Fraval A, Ming CY, Holcdorf D, Plunkett V, Tran P (2012). Internet use by orthopaedic outpatients - current trends and practices. Australas Med J.

[11] Baker JF, Devitt BM, Kiely PD, Green J, Mulhall KJ, Synnott KA, Poynton AR (2010). Prevalence of internet use amongst an elective spinal surgery outpatient population. Eur Spine J.

[12] Win KT, Hassan NM, Oinas-Kukkonen H, Probst Y (2016). Online patient education for chronic disease management: consumer perspectives. J Med Syst.

[13] Nguyen HQ, Carrieri-Kohlman V, Rankin SH, Slaughter R, Stulbarg MS (2004). Internet-based patient education and support interventions: a review of evaluation studies and directions for future research. Comput Biol Med.

[14] McKay HG, Glasgow RE, Feil EG, Boles SM, Barrera Jr MJ (2002). Internet-based diabetes self-management and support: initial outcomes from the Diabetes Network project. Rehabil Psychol.

[15] Ryhänen AM, Siekkinen M, Rankinen S, Korvenranta H, Leino-Kilpi H (2010). The effects of Internet or interactive computer-based patient education in the field of breast cancer: a systematic literature review. Patient Educ Couns.

[16] Hering K, Harvan J, D'Angelo M, Jasinski D (2005). The use of a computer website prior to scheduled surgery (a pilot study): impact on patient information, acquisition, anxiety level, and overall satisfaction with anesthesia care. AANA J.

[17] Wald HS, Dube CE, Anthony DC (2007). Untangling the Web--the impact of Internet use on health care and the physician-patient relationship. Patient Educ Couns.

[18] Idriss NZ, Alikhan A, Baba K, Armstrong AW (2009). Online, video-based patient education improves melanoma awareness: a randomized controlled trial. Telemed J E Health.

[19] Hungerford DS (2009). Internet access produces misinformed patients: managing the confusion. Orthopedics.

[20] Brooks BA (2001). Using the internet for patient education. Orthop Nurs.

[21] Cassidy JT, Baker JF (2016). Orthopaedic patient information on the World Wide Web: an essential review. J Bone Joint Surg Am.

[22] Jariwala AC, Kandasamy MS, Abboud RJ, Wigderowitz CA (2004). Patients and the internet: a demographic study of a cohort of orthopaedic out-patients. Surgeon.

[23] Roter DL, Hall JA, Merisca R, Nordstrom B, Cretin D, Svarstad B (1998). Effectiveness of interventions to improve patient compliance: a meta-analysis. Med Care.

[24] Feudtner C (2001). What are the goals of patient education?. West J Med.

[25] Schenker Y, Fernandez A, Sudore R, Schillinger D (2011). Interventions to improve patient comprehension in informed consent for medical and surgical procedures: a systematic review. Med Decis Making.

[26] Johansson K, Nuutila L, Virtanen H, Katajisto J, Salanterä S (2005). Preoperative education for orthopaedic patients: systematic review. J Adv Nurs.

[27] Ronco M, Iona L, Fabbro C, Bulfone G, Palese A (2012). Patient education outcomes in surgery: a systematic review from 2004 to 2010. Int J Evid Based Healthc.

[28] McDonald S, Page MJ, Beringer K, Wasiak J, Sprowson A (2014). Preoperative education for hip or knee replacement. Cochrane Database Syst Rev.

[29] Kroon FP, van der Burg LR, Buchbinder R, Osborne RH, Johnston RV, Pitt V (2014). Self-management education programmes for osteoarthritis. Cochrane Database Syst Rev.

[30] Louw A, Diener I, Butler DS, Puentedura EJ (2013). Preoperative education addressing postoperative pain in total joint arthroplasty: review of content and educational delivery methods. Physiother Theory Pract.

[31] Padilla GV, Bulcavage LM (1991). Theories used in patient/health education. Semin Oncol Nurs.

[32] Glanz K, Rimer BK, Viswanath K (2008). Health Behaviour and Health Education: Theory, Research, and Practice.

[33] Syx RL (2008). The practice of patient education: the theoretical perspective. Orthop Nurs.

[34] Moher D, Liberati A, Tetzlaff J, Altman DG, PRISMA group (2009). Preferred reporting items for systematic reviews and meta-analyses: the PRISMA statement. PLoS Med.

[35] Liberati A, Altman DG, Tetzlaff J, Mulrow C, Gøtzsche PC, Ioannidis JP, Clarke M, Devereaux PJ, Kleijnen J, Moher D (2009). The PRISMA statement for reporting systematic reviews and meta-analyses of studies that evaluate health care interventions: explanation and elaboration. PLoS Med.

[36] Barak A, Klein B, Proudfoot JG (2009). Defining internet-supported therapeutic interventions. Ann Behav Med.

[37] Downs SH, Black N (1998). The feasibility of creating a checklist for the assessment of the methodological quality both of randomised and non-randomised studies of health care interventions. J Epidemiol Community Health.

[38] Hootman JM, Driban JB, Sitler MR, Harris KP, Cattano NM (2011). Reliability and validity of three quality rating instruments for systematic reviews of observational studies. Res Synth Methods.

[39] Cindy Ng LW, Mackney J, Jenkins S, Hill K (2012). Does exercise training change physical activity in people with COPD? A systematic review and meta-analysis. Chron Respir Dis.

[40] Robbins SM, Houghton PE, Woodbury MG, Brown JL (2006). The therapeutic effect of functional and transcutaneous electric stimulation on improving gait speed in stroke patients: a meta-analysis. Arch Phys Med Rehabil.

[41] Eng JJ, Teasell R, Miller WC, Wolfe DL, Townson AF, Aubut J, et al (2007). Spinal Cord Injury Rehabilitation Evidence: methods of the SCIRE systematic review. Top Spinal Cord Inj Rehabil.

[42] Tranfield D, Denyer D, Smart P (2003). Towards a methodology for developing evidence-informed management knowledge by means of systematic review. Br J Management.

[43] Heikkinen K, Leino-Kilpi H, Vahlberg T, Salanterä S (2012). Ambulatory orthopaedic surgery patients’ symptoms with two different patient education methods. Int J Orthop Trauma Nurs.

[44] Heikkinen K, Helena LK, Taina N, Anne K, Sanna S (2008). A comparison of two educational interventions for the cognitive empowerment of ambulatory orthopaedic surgery patients. Patient Educ Couns.

[45] Heikkinen K, Leino-Kilpi H, Salanterä S (2012). Ambulatory orthopaedic surgery patients' knowledge with internet-based education. Methods Inf Med.

[46] Heikkinen K, Salanterä S, Leino-Kilpi H (2009). How do patients evaluate their education? - a comparison of two education methods. Stud Health Technol Inform.

[47] Heikkinen K, Salanterä S, Leppänen T, Vahlberg T, Leino-Kilpi H (2012). Ambulatory orthopaedic surgery patients' emotions when using different patient education methods. J Perioper Pract.

[48] Fraval A, Chandrananth J, Chong YM, Tran P, Coventry LS (2015). Internet based patient education improves informed consent for elective orthopaedic surgery: a randomized controlled trial. BMC Musculoskelet Disord.

[49] Yin B, Goldsmith L, Gambardella R (2015). Web-based education prior to knee arthroscopy enhances informed consent and patient knowledge recall: a prospective, randomized controlled study. J Bone Joint Surg Am.

[50] Groves ND, Humphreys HW, Williams AJ, Jones A (2010). Effect of informational internet web pages on patients' decision-making: randomised controlled trial regarding choice of spinal or general anaesthesia for orthopaedic surgery. Anaesthesia.

[51] Drieling RL, Ma J, Thiyagarajan S, Stafford RS (2011). An internet-based osteoporotic fracture risk program: effect on knowledge, attitudes, and behaviors. J Womens Health (Larchmt).

[52] Goldsmith DM, Safran C (1999). Using the web to reduce postoperative pain following ambulatory surgery. Proc AMIA Symp.

[53] Nahm ES, Barker B, Resnick B, Covington B, Magaziner J, Brennan PF (2010). Effects of a social cognitive theory-based hip fracture prevention web site for older adults. Comput Inform Nurs.

[54] Meesters JJL, de Boer IG, van den Berg MH, Fiocco M, Vliet Vlieland TP (2012). Evaluation of a website providing information on regional health care services for patients with rheumatoid arthritis: an observational study. Clin Rheumatol.

[55] Sobel D, Popp PL (2006). Informed consent and expectation management: a case study. J Healthc Risk Manag.

[56] Umapathy H, Bennell K, Dickson C, Dobson F, Fransen M, Jones G, Hunter DJ (2015). The web-based osteoarthritis management resource My Joint Pain improves quality of care: a quasi-experimental study. J Med Internet Res.

[57] Eltorai AE, Sharma P, Wang J, Daniels AH (2015). Most American Academy of Orthopaedic Surgeons' online patient education material exceeds average patient reading level. Clin Orthop Relat Res.

[58] Nassiri M, Bruce-Brand RA, O'Neill F, Chenouri S, Curtin PT (2014). Surfing for hip replacements: has the “internet tidal wave” led to better quality information. J Arthroplasty.

[59] Keller S (2014). How readable is the patient education material found on top-rated hospital web sites?. J Hosp Librariansh.

[60] Fredericks S, Martorella G, Catallo C (2015). A systematic review of web-based educational interventions. Clin Nurs Res.

[61] Wilkinson A, Whitehead L (2009). Evolution of the concept of self-care and implications for nurses: a literature review. Int J Nurs Stud.

[62] Irvine AB, Russell H, Manocchia M, Mino DE, Cox Glassen T, Morgan R, Gau JM, Birney AJ, Ary DV (2015). Mobile-web app to self-manage low back pain: randomized controlled trial. J Med Internet Res.

[63] Trudeau KJ, Pujol LA, DasMahapatra P, Wall R, Black RA, Zacharoff K (2015). A randomized controlled trial of an online self-management program for adults with arthritis pain. J Behav Med.

[64] Michie S, Richardson M, Johnston M, Abraham C, Francis J, Hardeman W, Eccles MP, Cane J, Wood CE (2013). The behavior change technique taxonomy (v1) of 93 hierarchically clustered techniques: building an international consensus for the reporting of behavior change interventions. Ann Behav Med.

[65] Lustria ML, Cortese J, Noar SM, Glueckauf RL (2009). Computer-tailored health interventions delivered over the Web: review and analysis of key components. Patient Educ Couns.

[66] Bessell TL, McDonald S, Silagy CA, Anderson JN, Hiller JE, Sansom LN (2002). Do internet interventions for consumers cause more harm than good? A systematic review. Health Expect.

[67] Thorne E, Mackenzie PD, Wilson M (2017). Expanding role of the internet in the orthopaedic outpatient setting. Bull Am Coll Surg.

[68] Baker JF, Green J, Synnott KA, Stephens MM, Poynton AR, Mulhall KJ (2013). Internet use in an orthopaedic outpatient population. Curr Orthop Pract.

[69] Walsh KP, Rehman S, Goldhirsh J (2014). Disparities in internet use among orthopedic outpatients. Orthopedics.

[70] Gutierrez N, Kindratt TB, Pagels P, Foster B, Gimpel NE (2014). Health literacy, health information seeking behaviors and internet use among patients attending a private and public clinic in the same geographic area. J Community Health.

[71] Rankinen S, Salanterä S, Heikkinen K, Johansson K, Kaljonen A, Virtanen H, Leino-Kilpi H (2007). Expectations and received knowledge by surgical patients. Int J Qual Health Care.

[72] Klemetti S, Leino-Kilpi H, Charalambous A, Copanitsanou P, Ingadottir B, Istomina N, Katajisto J, Unosson M, Zabalegui A, Valkeapää K (2016). Information and control preferences and their relationship with the knowledge received among European joint arthroplasty patients. Orthop Nurs.

[73] Ackerman IN, Bucknill A, Page RS, Broughton NS, Roberts C, Cavka B, Schoch P, Brand CA (2017). Preferences for disease-related education and support among younger people with hip or knee osteoarthritis. Arthritis Care Res (Hoboken).

[74] Berendsen AJ, de Jong GM, Schuling J, Bosveld HE, de Waal MW, Mitchell GK, van der Meer K, Meyboom-de Jong B (2010). Patient's need for choice and information across the interface between primary and secondary care: a survey. Patient Educ Couns.

[75] Fredericks S, Guruge S, Sidani S, Wan T (2010). Postoperative patient education: a systematic review. Clin Nurs Res.

