# Supplementary material for: Web-Based Patient Education in Orthopedics: Systematic Review
Source: J Med Internet Res. 2018 Apr 23;20(4):e143. doi: 10.2196/jmir.9013 (PMC5938597; doi:10.2196/jmir.9013)
Supplement: Multimedia Appendix 1 [file jmir_v20i4e143_app1.pdf]

Multimedia Appendix 1. Search strategies for the identification of studies assessing the effects of Web-based patient education interventions for the adult orthopedic population.

Table 1. Overall strategy for the identification of studies assessing the effects of Web-based patient education interventions for the adult orthopedic population.

	**Aspects**: combine with **AND**				
**Synonyms**: combine with **OR**	*Aspect 1: internet*	*Aspect 2: patient education*	*Aspect 3: orthopedic*	*Aspect 4: adult*	*Aspect 5: effectiveness*
	"World Wide Web"	"Medical Education"	orthopedic	adult	effect
	online	"Health Education"	orthopedics	aged	efficacy
	web-based	"Patient Education As A Topic"	orthopaedics		performance
	"Computer Assisted"	"Health Knowledge, Attitudes, Practice"	"joint replacement"		result
	E-Health	"Consumer Health Information"	"arthroplasty"		outcome
	network		hip		
	"Web Services"		knee		

Table 2. Specific strategies for the identification of studies assessing the effects of Web-based patient education interventions for the adult orthopedic population.

Database	Search string
Cochrane Central Register of Controlled Trials	("internet" or "world wide web" or "online" or "web-based" or "E-health") and ("patient education" or "health education" or "consumer health information") and (arthroplasty or orthopeadic or orthopedic or "joint replacement" or "hip replacement" or "knee replacement")<br>*with Bone, Joint and Muscle Trauma Group, Consumers and Communication Group or Musculoskeletal Group in Review Groups*
CINAHL	((MH "Arthroplasty, Replacement, Hip") OR (MH "Arthroplasty, Replacement, Knee") OR (MH "Arthroplasty, Replacement, Shoulder") OR (MH "Arthroplasty, Replacement, Ankle") OR (MH "Arthroplasty, Replacement") OR (MH "Arthroplasty, Replacement, Elbow") OR (MH "Arthroplasty, Knee, Unicompartmental") OR (MH "Arthroplasty, Reverse Total, Shoulder")) AND (((MH "Education, Health Information Management") OR (MH "Health Education") OR (MH "Patient Education")) AND (internet OR (MH "World Wide Web") OR (MH "World Wide Web Applications"))
EMBASE	(!effectiveness OR effect OR 'efficacy'/exp OR efficacy OR 'performance'/exp OR performance OR result OR 'outcome'/exp OR outcome) AND ((orthopaedic OR orthopedic OR orthopedics OR orthopaedics OR joint) AND replacement OR arthroplasty OR hip OR knee) AND ([adult]/lim OR [middle aged]/lim OR [aged]/lim OR [very elderly]/lim) AND ('patient education' OR 'medical education' OR 'health education' OR 'patient education as a topic' OR 'health knowledge, attitudes, practice' OR 'consumer health information') AND (internet OR 'world wide web' OR online OR 'web based' OR 'computer assisted' OR 'e health' OR network OR 'web services')
MEDLINE	((((TOPIC:((((("world wide web") OR online) OR web-based) OR "computer-assisted") OR E-health) OR "web services")AND TOPIC: (((("medical education") OR "health education") OR "Patient education") OR "Health knowledge, attitudes, practice") OR "consumer health information")) AND TOPIC:(((((orthopedic*) OR orthopeadic*) OR "joint replacement") OR "arthroplasty") OR hip) OR knee)) AND AGE GROUP: (((("Adult") OR "Middle Aged") OR "Aged") OR "Aged") OR "Aged, 80 and over")) AND TOPIC:((((effect) OR efficacy) OR performance) OR result) OR outcome))
PsycINFO	(Effectiveness or Effect or efficacy or performance or result or outcome).mp. [mp=title, abstract, full text, caption text] AND (adult or aged).mp. [mp=title, abstract, full text, caption text] AND (orthopaedic or orthopedic or orthopedics or orthopaedics or joint replacement or arthroplasty or hip or knee).mp. [mp=title, abstract, full text, caption text]

	AND ("patient education" or "medical education" or "health education" or "patient education as a topic" or "health knowledge, attitudes, practice" or "consumer health information").ab. AND (internet or "world wide web" or online or web-based or "computer assisted" or e-health or network or "web services").ab.
PubMed	(internet OR “world wide web” OR online OR web-based OR “computer assisted” OR e-health OR network OR “web services”) AND (“patient education” OR “patient education as topic” [MeSH Terms] OR “consumer health informati*” OR “medical education” OR “health education” OR “health knowledge, attitudes, practice”[MeSH Terms]) AND (orthopedic* OR orthopaedic* OR “joint replacement” or “arthroplasty” OR “hip” OR “knee”) AND (Adult OR Aged) AND (Effect OR efficacy OR performance OR result OR outcome)
Science direct	("internet" OR "world wide web" OR "online" OR "web-based" OR "E-health") AND ("patient education" OR "consumer health information") AND (arthroplasty OR orthopeadic OR orthopedic OR "joint replacement" OR "hip replacement" OR "knee replacement")
Scopus	((TITLE-ABS-KEY ("Effectiveness") OR TITLE-ABS-KEY ("Effect") OR TITLE-ABS-KEY (efficacy) OR TITLE-ABS-KEY (performance) OR TITLE-ABS-KEY ("result") OR TITLE-ABS-KEY ("outcome"))) AND ((TITLE-ABS-KEY ("patient education") OR TITLE-ABS-KEY ("medical education") OR TITLE-ABS-KEY ("Health Education") OR TITLE-ABS-KEY ("Patient Education As A Topic") OR TITLE-ABS-KEY ("Health Knowledge, Attitudes, Practice") OR TITLE-ABS-KEY ("Consumer Health Information"))) AND ((TITLE-ABS-KEY ("adult") OR TITLE-ABS-KEY ("aged"))) AND ((TITLE-ABS-KEY ("World Wide Web") OR TITLE-ABS-KEY ("Online") OR TITLE-ABS-KEY ("Web-Based") OR TITLE-ABS-KEY ("Computer Assisted") OR TITLE-ABS-KEY (e-health) OR TITLE-ABS-KEY (network) OR TITLE-ABS-KEY ("Web Services") OR TITLE-ABS-KEY (internet))) AND ((TITLE-ABS-KEY ("orthopedic") OR TITLE-ABS-KEY ("orthopedics") OR TITLE-ABS-KEY ("orthopaedics") OR TITLE-ABS-KEY ("joint replacement") OR TITLE-ABS-KEY ("arthroplasty") OR TITLE-ABS-KEY (hip) OR TITLE-ABS-KEY (knee) OR TITLE-ABS-KEY (orthopaedic)))
Web of Science	TS=("world wide web" OR online OR web-based OR "computer-assisted" OR e-health) AND TS=((((("medical education") OR "health education") OR "Patient education") OR "Health knowledge, attitudes, practice") OR "consumer health information") AND TS=(orthopedic* OR orthopeadic* OR "joint replacement" OR "arthroplasty" OR hip OR knee) AND TS=("Adult" OR "Middle Aged" OR "Aged" OR "Aged, 80 and over") AND TS=(((((effect) OR efficacy) OR performance) OR result) OR outcome)
