# Supplementary material for: Web-Based Patient Education in Orthopedics: Systematic Review
Source: J Med Internet Res. 2018 Apr 23;20(4):e143. doi: 10.2196/jmir.9013 (PMC5938597; doi:10.2196/jmir.9013)
Supplement: Multimedia Appendix 2 [file jmir_v20i4e143_app2.pdf]

Multimedia Appendix 2. Intervention characteristics of studies evaluating the effects of Web-based patient education in orthopedics (alphabetical order).

Authors, year, country	Name of intervention	Components in intervention [1]				Theoretical basis of intervention	Duration and frequency of intervention
		Program content	Multimedia use	Interactive online activities	Feedback support provision		
Drieling et al (2011), United States [2]	Bone Health Improvement Project	Tailored messages about risk factors, knowledge, attitudes, and behaviors.<br><br>Nontailored educational materials including recipes, exercise examples, and safety recommendations.	Text, graphics. Links to videos and other resources.	Tutorials include activities to facilitate behaviors, including problem-solving and goal-setting exercises.	Tailored automated feedback in the form of a personalized subset of intervention messages on the home page and in tailored tutorials.	Sender-message-channel-receiver communication model.<br>Social Cognitive Theory (SCT).<br>Transtheoretical model.	Intervention duration 6 months, 10 tailored tutorials (length 60-90 min) during first 3 months and 8 tailored tutorials during second 3 months.
Fraval et al (2015), Australia [3]	Orthoanswer	Walkthrough overview of procedures of diagnosis and indications for surgery; complications; and preoperative, intraoperative, and postoperative care. Frequently asked questions.	Text, graphics.	Not specified.	Not specified.	Not specified.	Duration not specified. Website visited once.

Goldsmith and Safran (1999), United States [4]	Ambulatory Surgery Nursing website	Information on surgery practicalities and discharge information. Password-protected access to pain management information section.	Not specified.	Not specified.	Not specified.	Not specified.	Not specified.
Groves et al (2010), United Kingdom [5]	Your Spinal Anaesthetic, the Health library, NHS Direct	Information about anesthesia, particularly with respect to total knee arthroplasty and total hip arthroplasty.	Text, graphics.	Not specified.	Not specified.	Not specified.	Not specified.
Heikkinen et al (2008; 2009; 2012a; 2012b; 2012c), Finland [6-10]	Not specified	Information about nine topics on surgery including instructions for preparing to the surgery, events of the surgery day, follow-up care, and financial aspects. Frequently asked questions.	Text, graphics, video.	Self-selection of the amount, depth and dimension of the knowledge provided by structuring information to knowledge level (basic, intermediate, and advanced).	Option to contact nurse via email or phone.	Cognitive empowerment.	Website use 10-300 min (mean=80.7, SD=66.7), 4-6 times (mean=2.3).
Meesters et al (2012), Netherlands [11]	Not specified	Information regarding practical aspects of health care services divided in six areas: “health care services,” “health professionals,”	Text, graphics.	Option to subscribe to a newsletter to receive the announcement of	Contact details of health services and health professionals. Telephone and	Not specified.	Website online for 30 months. Duration and frequency of visits not specified.

		“rheumatic diseases and physical activity,” “information and support,” “local consumer organization,” and “assistive devices”		relevant regional events.	email helpline to contact clinical nurse specialist.		
Nahm et al (2010), United States [12]	Social Cognitive Theory (SCT)– based, structured HPF website	Structured Web learning modules on osteoporosis, falls and hip fractures, dietary or supplementary calcium intake, and exercise. Patient testimonials.	Text, graphics, audio, animation, video,	Self-assessment quizzes.	Discussion board moderated by research nurse.	SCT.	2 weeks, 2 modules of 20-30 min per week.
Sobel and Popp (2006), United States [13]	EMMIPrep tool	Information about the surgical experience from preoperative to postoperative, including risks, benefits, and alternatives.	Text, animation, other unspecified learning modalities.	Option to type in questions.	Not specified.	Not specified.	Duration of online education 20 min. Frequency not specified.
Umapathy et al (2015), Australia [14]	My Joint Pain	Information for management of knee and hip osteoarthritis (OA) on treatment and management options and health care providers. Patient narratives (in video format).	Text, graphics, videos.	Hip or knee OA risk assessment. Monitoring of OA through weekly, monthly, and biannual pain assessment.	Tailored reinforcement messages based on assessment. Detailed report that could be discussed	Criteria for judging the quality of patient decision aids as specified by the International Patient Decision Aids	Website online for 12 months. Duration and frequency of visits not specified.

		Customized management plan based on assessment.			with health care team.	Standards Collaboration.	
Yin et al (2015), United States [15]	ExplainMySurgery.com, custom knee arthroscopy module	Explanation of relevant anatomy, pathology, and general perioperative instructions for knee arthroscopy.	Not specified.	Not specified.	Not specified.	Not specified.	20 min, once.

References

1. Barak A, Klein B, Proudfoot JG. Defining internet-supported therapeutic interventions. Ann Behav Med 2009 Aug; 38(1):4–17. PMID: 19787305
2. Drieling RL, Ma J, Thiyagarajan S, Stafford RS. An internet-based osteoporotic fracture risk program: effect on knowledge, attitudes, and behaviors. J Womens Health (Larchmt) 2011 Dec; 20(12):1895–1907. PMID: 21970565
3. Fraval A, Chandrananth J, Chong YM, Tran P, Coventry LS. Internet based patient education improves informed consent for elective orthopaedic surgery: a randomized controlled trial. BMC Musculoskelet Disord 2015 Feb 07; 16:14. PMID: 25885962
4. Goldsmith DM, Safran C. Using the Web to reduce postoperative pain following ambulatory surgery. Proc AMIA Symp 1999;780–4. PMID: 10566466
5. Groves ND, Humphreys HW, Williams AJ, Jones A. Effect of informational internet web pages on patients' decision-making: randomised controlled trial regarding choice of spinal or general anaesthesia for orthopaedic surgery. Anaesthesia 2010 Mar; 65(3):277–282. PMID: 20336817
6. Heikkinen K, Leino-Kilpi H, Vahlberg T, Salanterä S. Ambulatory orthopaedic surgery patients' symptoms with two different patient education methods. Int J Orthop Trauma Nurs 2012 Feb; 16(1):13–20. PMID: 22476362
7. Heikkinen K, Helena LK, Taina N, Anne K, Sanna S. A comparison of two educational interventions for the cognitive empowerment of ambulatory orthopaedic surgery patients. Patient Educ Couns 2008 Nov; 73(2):272–279. PMID: 18678461

8. Heikkinen K, Leino-Kilpi H, Salanterä S. Ambulatory orthopaedic surgery patients' knowledge with internet-based education. Methods Inf Med 2012; 51(4):295–300. PMID: 22476362

9. Heikkinen K, Salanterä S, Leino-Kilpi H. How do patients evaluate their education? - a comparison of two education methods. Stud Health Technol Inform 2009; 146:850–1. PMID: 19593017

10. Heikkinen K, Salanterä S, Leppänen T, Vahlberg T, Leino-Kilpi H. Ambulatory orthopaedic surgery patients' emotions when using two different patient education methods. J Perioper Pract 2012 Jul; 22(7):226–31. PMID: 22919767

11. Meesters JJL, De Boer IG, Van Den Berg MH, Fiocco M, Vliet Vlieland TPM. Evaluation of a website providing information on regional health care services for patients with rheumatoid arthritis: an observational study. Clin Rheumatol 2012 Apr; 31(4):637–45. PMID: 22160606

12. Nahm ES, Barker B, Resnick B, Covington B, Magaziner J, Brennan PF. Effects of a social cognitive theory-based hip fracture prevention web site for older adults. Comput Inform Nurs 2010 Nov; 28(6):371–9. PMID: 20978408

13. Sobel D, Popp PL. Informed consent and expectation management: a case study. J Healthc Risk Manag 2006; 26(4):21–26. PMID: 19606763

14. Umapathy H, Bennell K, Dickson C, Dobson F, Fransen M, Jones G, Hunter DJ. The web-based osteoarthritis management resource My Joint Pain improves quality of care: a quasi-experimental study. J Med Internet Res 2015 Jul 07; 17(7):e167. PMID: 26154022

15. Yin B, Goldsmith L, Gambardella R. Web-based education prior to knee arthroscopy enhances informed consent and patient knowledge recall: a prospective, randomized controlled study. J Bone Jt Surg Am 2015 Jun 17; 97(12):964–71. PMID: 26085529
