# Supplementary material for: Web-Based Patient Education in Orthopedics: Systematic Review
Source: J Med Internet Res. 2018 Apr 23;20(4):e143. doi: 10.2196/jmir.9013 (PMC5938597; doi:10.2196/jmir.9013)
Supplement: Multimedia Appendix 3 [file jmir_v20i4e143_app3.pdf]

Multimedia Appendix 3. Summary of the effects of Web-based patient education (ordered by comparison then by quality).

Authors	Design	Quality assessment	Patient population	Intervention, sample size (n), age (years)	Control, sample size (n), age (years)	Timing of outcome measures	Outcome measures, instruments	Intervention effects[a]	Intervention effects relative to control[a]
No comparison									
Umapathy et al [1]	Quasi-experimental study	High	Patients with self-assessed hip or knee osteoarthritis<br><br>N=195	Online tailored information tool,<br><br>n=104, age=mean 60.9 (SD 9.1)	Nonusers,<br><br>n=91, age=mean 60.5 (SD 8.3)	Pre test, post test (12 months)	Knowledge	=[b]	=
							Self-management	+	=
Meesters et al [2]	Observational study	Medium	Patients with rheumatoid arthritis<br>N=400 (160 paired observations analyzed)	Informational website, n=160, age=mean 60.4 (SD 9.9)	Not applicable (N/A)	Pre test, post test (24 months)	Knowledge insufficiency	−	N/A
							Information needs	−	N/A

Sobel and Popp [3]	Retrospective survey analysis	Low	Patients undergoing total hip arthroplasty (THA) and other surgeries (gastric bypass, colonoscopy)<br><br>N=2423	Online education tool, n=2423, age not reported	N/A	Post test	Knowledge (general)	+	N/A
							Knowledge (risks)	+	N/A
							Confidence in provider	+	N/A
							Perceived usefulness	Positive evaluation	N/A
Comparison to health information websites									
Drieling et al [4]	Pilot Randomized Controlled Trial (RCT)	High	Healthy females at risk of osteoporosis N=121	Internet-based risk- and stage-tailored health education program, n=61,	Independent review of online health information, n=60,	Pre test, post test (3 months), follow-up (6 months)	Knowledge (osteoporosis)	×	+[d]
							Knowledge (calcium)	×	+

				age=mean 59.9 (SD 6.7)[c]	age=mean 58 (SD 8.6)[c]		Knowledge (exercise)	×	=
							Self-efficacy	×	=
							Beliefs	×	=
							Exercise	×	=
							Calcium intake	×	=
							Vitamin D intake	×	=
Nahm et al [5]	RCT	High	Older adults from general population at risk of hip fracture N=245	SCT-based structured hip fracture prevention website, n=125,	Conventional hip fracture prevention website, n=120,	Pre test, post test (2 weeks), follow-up (3 months)	Knowledge (hip fracture)	+	=
							Knowledge (osteoporosis)	+	=

				age=mean 69.3 (SD 7.7)[e]	age=mean 69.3 (SD 7.7)[e]		Self-efficacy (calcium intake)	+	=
							Self-efficacy (exercise)	=	=
							Self-efficacy (online learning)	×	−
							Expectations (calcium intake)	+	=
							Expectations (exercise)	=	=
							Calcium intake	+	=
							Exercise	=	=

							Satisfaction	×	+
Goldsmith and Safran [6]	Prospective RCT	Medium	Patients scheduled for preoperative screening for orthopedic, gynecological, and laparoscopic ambulatory surgery<br><br>N=195	Preoperative interview + informational website + password-protected access to pain management section, n=98, age=mean 45.2 (range: 19-82)	Preoperative interview + limited access to information website (n=97, age=mean 44.5 (range: 18-74)	Post test (arrival at home), 1st follow-up (night after surgery), 2nd follow-up (day after surgery)	Pain	×	−
							Utilization	×	+
							Perceived usefulness	+	×
Comparison to traditional patient education									
Fraval et al [7]	RCT	High	Orthopedic outpatients scheduled for five common orthopedic	Standard verbal consent discussion + online patient education tool,	Standard verbal consent discussion, n=108, age=mean53.7	Post test	Knowledge	×	+

			procedures: total knee arthroplasty (TKA), THA, knee arthroscopy, shoulder arthroscopy, and anterior cruciate ligament reconstruction<br><br>N=211	n=103, age=mean 54.29 (SD not reported)	(SD not reported)		Anxiety	×	=
							Satisfaction	×	+
Groves et al [8]	Double-blind RCT	High	Patients presenting to the orthopedic preadmission clinic before admission for TKA or THA<br><br>N=118	Written patient information sheet + online health information websites, n=59, age=mean 58.7 (SD 10.4)	Written patient information sheet, n=59, age= mean 62 (SD 9)	Pre test, post test (at admission for surgery or 2 weeks after clinic appointment)	Knowledge	+	+
							Choice for neuraxial	+	+
Yin et al [9]	Prospective RCT	High	Adult patients undergoing first-	Standard verbal preoperative	Standard verbal preoperative	Post test (preoperative	Knowledge	×	+

			time knee arthroscopy for primary diagnosis of meniscal tear<br><br>N=55	education and counseling with surgeon + Web-based tutorial, n=26, age=mean 49.9 (SD 10.3)	education and counseling with surgeon, n=29, age=mean 47.5 (SD 13.3)	visit), 1st follow-up (day of surgery), 2nd follow-up (1st postoperative visit)[f]	Knowledge sufficiency	×	+
							Anxiety	×	=[g]
							Satisfaction	×	+
							Perceived usefulness	Positive evaluation	×
Heikkinen et al[h] [10]	RCT	High	Ambulatory orthopedic surgery (shoulder or knee arthroscopy) patients<br>N=149	Web-based education via website, n=72, age=mean 44.2 (SD 12.7)	Verbal education with nurse, n=75, age=mean (SD 12.7)	Pre test, post test, follow-up (2 weeks after surgery)	Knowledge (overall, functional, ethical)	+	+
							Knowledge (bio-physiological, experiential, social, financial)	+	=

							Knowledge sufficiency (ethical)	+	+
							Knowledge sufficiency (overall, bio-physiological, functional, experiential, social, financial)	+	=
Heikkinen et al[h] [11]	-	High	-	-	-	Pre test, post test, follow-up (surgery day, 1st and 3rd postoperative day, 2 and 4 weeks postoperative)	Emotions	=	=

Heikkinen et al[h] [12]	-	High	-	-	-	Pre test, post test, follow-up (2 weeks after surgery)	Knowledge	+	+
							Knowledge sufficiency	+	=
Heikkinen et al[h] [13]	-	High	-	-	-	Pre test, post test, follow-up (surgery day, 1st and 3rd postoperative day, 2 and 4 weeks postoperative)	Pain (symptoms)	=	=
							Function (symptoms)	+	=
Heikkinen et al[h] [14]	-	Medium	-	-	-	Post test	Information needs	×	=
							Understanding	×	=

							Self-efficacy	×	−
							Anxiety	×	+
							Clarity of content	×	−
							Satisfaction	×	=

[a]Statistically significant results: (+), positive effect; (=), not significant; (−), negative effect; (×), not reported.

[b]Although overall knowledge improvement was not significant, it did significantly improve for subscales self-management, lifestyle, and physical activity (+).

[c]To increase ease of comparison, mean age and SD were approximated from the frequency tables reported in the original studies.

[d]Change from baseline to 6 months was significant; however, change from baseline to 3 months was not significant.

[e]Mean age and SD were not provided separately for each group. The mean age and SD of the overall sample is reported.

[f]Measures from post test to follow-up were rephrased over the study period.

[g]Change was significant at 2nd follow-up; however, change at post test or 1st follow-up was not significant.

[h]All papers by Heikkinen and colleagues report different outcome measures following the same intervention. As such, the study design, intervention group, control group, and sample sizes as described for Heikkinen et al (2008) [10] are identical to [11-14].

References

1. Umapathy H, Bennell K, Dickson C, Dobson F, Fransen M, Jones G, Hunter DJ. The web-based osteoarthritis management resource My Joint Pain improves quality of care: a quasi-experimental study. J Med Internet Res 2015 Jul 07; 17(7):e167. PMID: 26154022

2. Meesters JJL, De Boer IG, Van Den Berg MH, Fiocco M, Vliet Vlieland TPM. Evaluation of a website providing information on regional health care services for patients with rheumatoid arthritis: an observational study. Clin Rheumatol 2012 Apr; 31(4):637–45. PMID: 22160606

3. Sobel D, Popp PL. Informed consent and expectation management: a case study. J Healthc Risk Manag 2006; 26(4):21–26. PMID: 19606763

4. Drieling RL, Ma J, Thiyagarajan S, Stafford RS. An internet-based osteoporotic fracture risk program: effect on knowledge, attitudes, and behaviors. J Womens Health (Larchmt) 2011 Dec; 20(12):1895–1907. PMID: 21970565

5. Nahm ES, Barker B, Resnick B, Covington B, Magaziner J, Brennan PF. Effects of a social cognitive theory-based hip fracture prevention web site for older adults. Comput Inform Nurs 2010 Nov; 28(6):371–9. PMID: 20978408

6. Goldsmith DM, Safran C. Using the Web to reduce postoperative pain following ambulatory surgery. Proc AMIA Symp 1999;780–4. PMID: 10566466

7. Fraval A, Chandrananth J, Chong YM, Tran P, Coventry LS. Internet based patient education improves informed consent for elective orthopaedic surgery: a randomized controlled trial. BMC Musculoskelet Disord 2015 Feb 07; 16:14. PMID: 25885962

8. Groves ND, Humphreys HW, Williams AJ, Jones A. Effect of informational internet web pages on patients' decision-making: randomised controlled trial regarding choice of spinal or general anaesthesia for orthopaedic surgery. Anaesthesia 2010 Mar; 65(3):277–282. PMID: 20336817

9. Yin B, Goldsmith L, Gambardella R. Web-based education prior to knee arthroscopy enhances informed consent and patient knowledge recall: a prospective, randomized controlled study. J Bone Jt Surg Am 2015 Jun 17; 97(12):964–71. PMID: 26085529

10. Heikkinen K, Helena LK, Taina N, Anne K, Sanna S. A comparison of two educational interventions for the cognitive empowerment of ambulatory orthopaedic surgery patients. Patient Educ Couns 2008 Nov; 73(2):272–279. PMID: 18678461

11. Heikkinen K, Salanterä S, Leppänen T, Vahlberg T, Leino-Kilpi H. Ambulatory orthopaedic surgery patients' emotions when using two different patient education methods. J Perioper Pract 2012 Jul; 22(7):226–31. PMID: 22919767

12. Heikkinen K, Leino-Kilpi H, Salanterä S. Ambulatory orthopaedic surgery patients' knowledge with internet-based education. Methods

Inf Med 2012; 51(4):295–300. PMID: 22476362

13. Heikkinen K, Leino-Kilpi H, Vahlberg T, Salanterä S. Ambulatory orthopaedic surgery patients' symptoms with two different patient education methods. Int J Orthop Trauma Nurs 2012 Feb; 16(1):13–20. PMID: 22476362

14. Heikkinen K, Salanterä S, Leino-Kilpi H. How do patients evaluate their education? - a comparison of two education methods. Stud Health Technol Inform 2009; 146:850–1. PMID: 19593017
